# Uniparental ancestry markers in Chilean populations

**DOI:** 10.1590/1678-4685-GMB-2015-0273

**Published:** 2016-08-04

**Authors:** Camilla Dutra Vieira-Machado, Maluah Tostes, Gabrielle Alves, Julio Nazer, Liliana Martinez, Elisabeth Wettig, Oscar Pizarro Rivadeneira, Marcela Diaz Caamaño, Jessica Larenas Ascui, Pedro Pavez, Maria da Graça Dutra, Eduardo Enrique Castilla, Ieda Maria Orioli

**Affiliations:** 1 Latin American Collaborative Study of Congenital Malformations (ECLAMC) and National Institute of Population Medical Genetics (INAGEMP), Departmento de Genética, Instituto de Biologia, Universidade Federal do Rio de Janeiro (UFRJ), Rio de Janeiro, RJ, Brazil; 2 Neonatal Service, Department of Obstetrics and Gynecology, Hospital Clínico de La Universidad del Chile, Santiago, Chile; 3 Hospital Regional de Valdivia, Valdívia, Chile; 4 Neonatology Service, Hospital Puerto Montt, Puerto Montt, Chile; 5 Hospital del Salvador, Santiago, Chile; 6 Hospital Clínico San Borja-Arriarán, Santiago, Chile; 7 Hospital de Cauquenes, Cauquenes, Chile; 8 Hospital Curicó, Curicó, Chile; 9 Latin American Collaborative Study of Congenital Malformations (ECLAMC) and National Institute of Population Medical Genetics (INAGEMP), Laboratory of Congenital Malformations Epidemiology (LEMC), Instituto Oswaldo Cruz (IOC), Fundação Oswaldo Cruz (Fiocruz), Rio de Janeiro, RJ, Brazil; 10 Latin American Collaborative Study of Congenital Malformations (ECLAMC) Center for Medical Education and Clinical Research (CEMIC) Buenos Aires, Argentina

**Keywords:** Uniparental markers, ancestry, mtDNA, Y-chromosome, ECLAMC

## Abstract

The presence of Native Americans, Europeans, and Africans has led to the development of a multi-ethnic, admixed population in Chile. This study aimed to contribute to the characterization of the uniparental genetic structure of three Chilean regions. Newborns from seven hospitals in Independencia, Providencia, Santiago, Curicó, Cauquenes, Valdívia, and Puerto Montt communes, belonging to the Chilean regions of Santiago, Maule, and Los Lagos, were studied. The presence of Native American mitochondrial DNA (mtDNA) haplogroups and two markers present in the non-recombinant region of the Y chromosome, DYS199 and DYS287, indicative of Native American and African ancestry, respectively, was determined. A high Native American matrilineal contribution and a low Native American and African patrilineal contributions were found in all three studied regions. As previously found in Chilean admixed populations, the Native American matrilineal contribution was lower in Santiago than in the other studied regions. However, there was an unexpectedly higher contribution of Native American ancestry in one of the studied communes in Santiago, probably due to the high rate of immigration from other regions of the country. The population genetic sub-structure we detected in Santiago using few uniparental markers requires further confirmation, owing to possible stratification for autosomal and X-chromosome markers.

The European conquest brought significant changes to the population of America, resulting in cultural and genetic exchange with the Native American and African populations.

Several studies have linked the matrilineage of Native American population to mitochondrial DNA (mtDNA) haplogroups A through D. These haplogroups were widespread in the Americas, while haplogroup X was restricted to North America (Torroni *et al.*, 1993; Bailliet *et al.*, 1994; Brown *et al.*, 1998). Subsequent studies using higher-resolution techniques, such as sequencing of the control region or the whole mtDNA, allowed the identification of sub-haplogroups related to each main haplogroup. So far, 10 monophyletic Pan-American sub-haplogroups have been identified (Achilli *et al.*, 2008; Perego *et al.*, 2010), each consisting of several, previously identified lineages.

In admixed populations, matrilineages of only Antofagasta (Henríquez *et al.*, 2004), Los Lagos (Garcia *et al.*, 2006), and Santiago Metropolitan (Rocco *et al.*, 2002) regions were investigated. Both, Antofagasta and Los Lagos regions showed a greater contribution of Native American ancestry by the matrilineage compared to that seen in the population of the Santiago metropolitan region. Studies using autosomal markers also showed the pattern of a smaller contribution of Native American ancestry in Central Chile when compared to the North and South of the country (Ruiz-Linares *et al.*, 2014; Eyheramendy *et al.*, 2015). Regarding patrilineage, most Native Americans belong to haplogroup Q, particularly to sub-haplogroup Q-M3 (Q1a3a) (Karafet *et al.*, 2008), which is found in all Native American populations. A C > T transition in the DYS199 locus, also named M3, identifies the sub-haplogroup Q-M3, found outside America only in Siberia, probably reflecting reverse gene flow from Alaska into Asia (Roewer *et al.*, 2013). Since there is no evidence to show that the M3 transition occurred more than once during human evolution, all Q-M3 haplogroups are believed to descend from a common ancestor wherein this transition occurred (Underhill *et al.*, 1996). Therefore, this marker is particularly useful for the identification of Y-chromosome haplotypes originating after migration to the American continent.

In addition to haplogroup Q, only haplogroup C is known to occur in Native American populations. In South America, the C3* paragroup was found only in Native populations from Ecuador (Zegura *et al.*, 2004; Roewer *et al.*, 2013). Other Y-chromosome haplogroups, such as haplogroup R, were probably introduced in America through admixture after the colonization of the continent by Europeans (Zegura *et al.*, 2004).

Limited data exists regarding the patrilineage of Chilean native and admixed populations. Chilean Pehuenches have high frequency of the haplogroup Q-M3, while Huilliches present a lower frequency of this haplogroup, probably owing to a greater contribution of the haplogroup R, indicative of European gene flow (Bailliet *et al.*, 2011).

Of all Chilean regions, only the admixed population of the Santiago metropolitan region has been studied, focusing on its paternal origin and diversity (Cifuentes *et al.*, 2004). Low frequencies of haplogroup Q-M3 were observed in both low and high socioeconomic strata samples, suggesting that the main contribution to the patrilineage of this population is European (Cifuentes *et al.*, 2004).

DYS287 is a 300-bp Alu-insertion polymorphism (Y Alu Polymorphism; YAP) with high frequency in African populations, reaching 95% in South and West Africa. Although it is also found in some Asian populations, its frequency in Africa is significantly higher than in any other region worldwide. It is believed that the YAP insertion occurred only once in Africa and that all Y-chromosomes carrying the YAP+ allele descended from this single individual (Hammer, 1994). YAP+ allele characterizes the haplogroup DE, showing very low frequencies in most native and admixed populations of America, which is considered evidence of admixture (Bravi *et al.*, 2000).

The aim of this study was to contribute to the characterization of the uniparental genetic structure of seven communes from Santiago Metropolitan, Maule, and Los Lagos regions of Chile.

We analyzed umbilical cord blood obtained anonymously from 659 consecutive births from each of the seven Chilean hospitals located in Independencia, Providencia, Santiago, Curicó, Cauquenes, Valdívia, and Puerto Montt communes, between 2000 and 2006. The number of newborns in each hospital and the location of each Chilean hospital is shown in Table 1. All hospitals belong to the Latin American Congenital Malformation Collaborative Study (ECLAMC) network (Castilla and Orioli, 2004) dedicated to the study of the causes of birth defects. The ECLAMC study protocol was approved by the Comité de Ética en Investigación del Centro de Educación Médica e Investigaciones Clínicas (Dr. Norberto Quirno) in Buenos Aires, Argentina (IRB-1745, IORG-0001315; approval number: #238). The samples used in this study were collected with the purpose of representing the population born at each hospital and serve as control in molecular studies.

**Table 1:** Hospitals from each Chilean commune, their geographical location and sample size.

Hospital	ECLAMC Code	Geographical Location^1^	Commune	Province	Region	No. of Samples
Hospital Clínico de la Univ. del Chile J.J. Aguirre	201	33° 25' S 70° 39' W	Independencia	Santiago	Santiago Metropolitan	76
Hospital del Salvador	222	33° 26' S 70° 37' W	Providencia	Santiago	Santiago Metropolitan	99
Hospital Clínico San Borja-Arriarán	223	33° 27' S 70° 38' W	Santiago	Santiago	Santiago Metropolitan	93
Hospital Curicó	227	34° 59' S 71° 14' W	Curicó	Curicó	Maule	96
Hospital de Cauquenes	226	35° 57' S 72° 19' W	Cauquenes	Cauquenes	Maule	96
Hospital Base Regional de Valdivia	205	39° 49' S 73° 14' W	Valdivia 2	Valdivia	Los Lagos	100
Hospital Base de Puerto Montt	220	41° 27' S 72° 56' W	Puerto Montt	Llanquihue	Los Lagos	99

1 Latitude and longitude in degrees and minutes

2 Valdivia is the capital of the Valdivia province in Los Rios region (14^th^ Chilean region), split from the Los Lagos region in 2007, however, as the sample was collected prior to that date, it will be considered as still part of Los Lagos.

Samples were genotyped for Native American mtDNA haplogroups as previously described by Bailliet *et al.* (1994). The mitochondrial lineages A, C, and D were identified by Restriction Fragment Length Polymorphism (RFLP) at positions 663, 13262, and 5178bp, respectively, and lineage B was identified by an intergenic deletion between COII-tRNA(Lys).

Samples not belonging to any Native American A-D mtDNA haplogroups were included in a group called "Other".

All samples studied were donated anonymously; therefore, there is no information about the newborns' sex. A molecular determination of sex was made in order to select only male samples for Y-chromosome analysis. This sex screening was performed as previously described by Nakahori *et al.* (1991).

DYS199 genotyping was performed as previously described by Santos *et al.* (1999) using a modified primer to create an artificial restriction site to *Mfe*I endonuclease in samples carrying the DYS199C allele. DYS287 was genotyped as previously described by Hammer and Horai (1995) only in DYS199C individuals, since the YAP+ allele is not found in chromosomes carrying the DYS199T allele (Karafet *et al.*, 1997).

Initially, the frequency in each commune was calculated for mtDNA haplogroups A-D and "Other," DYS199C and DYS199T alleles, and YAP+ and YAP- alleles in DYS287 locus.

Subsequently, to test the hypothesis of no difference among the communes, the chi-square test for homogeneity (BioEstat 5.0) was used to compare the frequencies of each Native American mtDNA haplogroup, combined Native American mtDNA haplogroups (A+B+C+D), and each allele of both Y-chromosome markers. Alpha error was set at 5%.

Genetic distance between communes was estimated by the fixation index (F_ST_) using Arlequin v3.5.1.3 software, with a significance test for 1023 permutations and alpha error set at 5%. To determine whether a haplogroup is responsible for genetic differentiation between communes, we calculated the F_ST_ value for each haplogroup using GENEPOP 4.13 software. Analysis of molecular variation (AMOVA) (Excoffier *et al.*, 1992) was also performed using Arlequin v3.5.1.3 software and the degree of subdivision was assessed, where communes were grouped by region (Table 1): Santiago Metropolitan, Maule, and Los Lagos.

All the studied communes showed contribution of Native American and non-Native American ancestry to the matrilineage and patrilineage (Tables 2, 3, and 4). Table 2 shows the frequencies of Native American mtDNA haplogroups A-D and of the group "Other," with high contribution of Native American ancestry to the matrilineage in all studied communes, ranging from 75% to 95%. This proportion of Native American ancestry was significantly different among communes (χ^2^ = 28.71, DF = 6, *p* < 0.0001). Independencia and Santiago communes in the Santiago Metropolitan region showed a non-Native American matrilineal contribution that was significantly higher than in the other studied communes, approaching 20% in the Santiago commune and 25% in the Independencia commune. The other communes showed an average rate of 8.4% non-Native American mtDNA haplogroups. A chi-square test of homogeneity excluding data from Independencia and Santiago communes showed no difference in the frequency of the Native American mtDNA haplogroup among the other five communes (χ2 = 7.35, DF = 4, *p* = 0.1186). This indicates that Independencia and Santiago populations are significantly different from the other populations in this study, regarding this contribution. They were also significantly different from the Providencia commune located in the same Santiago Metropolitan region (χ^2^ = 11.18, DF = 2, *p* = 0.0037), which is indicative of a population genetic sub-structure within the Santiago Metropolitan region.

**Table 2:** Native American mtDNA haplogroups in the seven Chilean communes.

Commune (Region)	N	Mitochondrial Haplogroups				Native American Total	%	95% Confidence Interval	Other	%	95% confidence Interval
		A	B	C	D	A+B+C+D					
Independência (Santiago MR)^1^	76	3	24	16	14	57	75.0	65.3 – 84.7	19	25.0	15.3 – 34.7
Providencia (Santiago MR) 1	99	8	33	26	25	92	92.9	87.8 – 98.0	7	7.1	2.0 – 12.2
Santiago (Santiago MR) 1	93	3	24	24	24	75	80.6	72.6 – 88.6	18	19.4	11.4 – 27.4
Curicó (Maule)	96	4	13	32	33	82	85.4	78.3 – 92.5	14	14.6	7.5 – 21.6
Cauquenes (Maule)	96	6	22	28	31	87	90.6	84.8 – 96.4	9	9.4	3.6 – 15.2
Valdivia (Los Lagos)	100	1	25	37	32	95	95.0	90.7 – 99.3	5	5.0	0.7 – 9.3
Puerto Montt (Los Lagos)	99	5	23	30	35	93	93.9	89.2 – 98.6	6	6.1	1.4 – 10.8
Total	659	30	164	193	194	581	88.2	85.7 – 90.6	78	11.8	9.3 – 14.2

1 Santiago Metropolitan Region.

**Table 3:** DYS199T allele frequency in the seven Chilean communes

Commune (Region)	N	DYS199T	%	95% Confidence Interval
Independência (Santiago MR) 1	40	3	7.5	0 – 15.6
Providencia (Santiago MR) 1	39	5	12.8	2.3 – 23.3
Santiago (Santiago MR) 1	43	5	11.6	2.0 – 21.2
Curicó (Maule)	51	4	7.8	0.5 – 15.2
Cauquenes (Maule)	50	3	6.0	0.6 – 12.6
Valdivia (Los Lagos)	44	4	9.0	0.5 – 17.5
Puerto Montt (Los Lagos)	37	2	5.4	0.0 – 12.7
Total	304	26	8.5	5.4 – 11.6

1 Santiago Metropolitan Region.

**Table 4:** YAP+ allele frequency in the seven Chilean communes

Commune (Region)	N	YAP+	%	95% Confidence Interval
Independência (Santiago MR) 1	40	4	10.0	0.7 – 19.3
Providencia (Santiago MR) 1	39	5	12.8	2.3 – 23.3
Santiago (Santiago MR) 1	40	4	10.0	0.7 – 19.3
Curicó (Maule)	50	7	14.0	4.4 – 23.6
Cauquenes (Maule)	50	4	8.0	0.5 – 15.5
Valdivia (Los Lagos)	44	1	2.3	0.0 – 6.7
Puerto Montt (Los Lagos)	37	1	2.7	0.0 – 7.9
Total	300	26	8.7	5.5 – 11.9

1 Santiago Metropolitan Region.

The proportions of each Native American mtDNA haplogroups were not significantly different among communes (χ2 = 24.69, DF = 18, p = 0.134), with higher frequencies of haplogroups D (33.4%), C (33.2%), and B (28.2%), whereas a lower frequency of haplogroup A (5.2%).

Table 3 shows the frequencies of DYS199T allele in the seven Chilean communes. Unlike that observed for mtDNA, the chi-square test showed no significant difference for the DYS199 SNP allele frequencies among communes (χ^2^ = 2.42, DF = 6, *p*=0.877).

As shown in Table 4, the YAP insertion average frequency was at 8.7%, showing low contribution of African ancestry in the patrilineage, as previously observed for the Native American ancestry estimated by DYS199 locus. There was also no significant difference in the frequency of this insertion among the studied communes (χ^2^ = 7.18, DF = 6, *p*=0.3042).

Our results indicate a noticeably smaller patrilineal (8.5%) than matrilineal contribution (88.2%) of Native American ancestry in terms of Native American mtDNA haplogroups (Table 2). This pattern of a significantly larger contribution of Native American ancestry from the matrilineage than from the patrilineage is consistent with that reported in previous studies from South America (Martinez-Marignac *et al.*, 2004; Bonilla *et al.*, 2005; Vieira-Machado CD, 2011, Dissertation, Universidade Federal do Rio de Janeiro). This phenomenon is attributed to marriages that took place between European men and Native American women during the colonization of the American continent (Ruiz-Linares, 2014).

The lower contribution of Native American ancestry to the Independencia and Santiago communes as compared to the other regions is in agreementwith previous reports involving autosomal (Fuentes *et al.*, 2014; Eyheramendy *et al.*, 2015) and mtDNA markers (Rocco *et al.*, 2002; Henríquez *et al.*, 2004; Garcia *et al.*, 2006). These studies showed a lower contribution of Native American ancestry in the central regions of the country, probably a result of a higher immigration rate from Spain during the colonization of Chile and from several other European regions after 1850.

A population sub-structure has been reported between populations that inhabited the Ayllus (Andean kin-based community structure) of the San Pedro de Atacama's oases (Northern Chile) during the Middle Period (AD 400-1000), by means of nonmetric cranial traits of skeletal remains from 12 cemeteries. This finding suggested that differential migration and/or cultural isolation processes accentuated biological differences between the Ayllus (Torres-Rouff *et al.*, 2013). Since there is no evidence of isolation among the studied communes of Santiago Metropolitan region, the difference in the Native American mtDNA contribution was probably a consequence of the high rate of immigration of individuals with higher Native American ancestry from other regions of the country during the 1960s (Villa and Rodríguez, 1996). The heterogeneous genetic composition of Santiago Metropolitan region has not been observed previously.

Marcheco-Teruel *et al.* (2014), studied population samples from all Cuban provinces by means of autosomal and uniparental markers, and showed that uniparental markers identified the same differences between provinces that had been identified previously using autosomal markers. Therefore, the heterogeneity found among the communes in the Santiago Metropolitan region in the present study probably may be true also for the autosomal and X-chromosome markers.

Regarding the patrilineage, our results showed a homogeneous contribution of Native American and African ancestries to the three studied regions with low frequencies of both ancestries in all studied communes. The lower frequency for the DYS199T allele, ranging from 5.4% to 12.8%, showed little Native American patrilineal contribution to the studied communes, similar to previous findings in admixed population from the Santiago Metropolitan region (4.3%) (Cifuentes *et al.*, 2004). Similar frequencies have been observed in other admixed American populations like those in La Plata, Argentina (9.4%) (Martinez-Marignac *et al.*, 2004) and Belém, northern Brazil (3.8%) (Batista dos Santos *et al.*, 1999), while higher frequencies (44.0%) were found in Pasco and Lima, Peru (Rodriguez-Delfin *et al.*, 2001) and in the Jujuy Province, northwestern Argentina (43.7% up to 94.7%) (Bailliet *et al.*, 2011).

The YAP+ allele of the DYS287 locus, the other studied marker present in the non-recombinant region of the Y chromosome, is found in Africa with frequencies ranging from 49.5% in the northern region to up to 95.0% in Sub-Saharan Africa. In Japan, its frequency ranges from 33.0% to 56.0%, probably owing to its inheritance from their Jomon ancestors, who remained isolated for 13,000 years, suffering from genetic drift effects, which might have led to the increase in YAP+ allele frequency in that population (Hammer and Horai, 1995). In the admixed populations of America, YAP+ allele frequency ranged from low values observed in Pasco and Lima, Peru (4.0%) (Rodriguez-Delfin *et al.*, 2001) and in different Argentinean regions, ranging from 1% in Jujuy Province to 20% in Salta Province (Bailliet *et al.*, 2011), up to 40.5% in Cartagena, an admixed population in Colombian Caribbean region (Rojas *et al.*, 2010). Only the Chilean Native populations Huilliche and Pehuenche were investigated for the presence of YAP+ or DE haplogroup both showing very low frequencies of this marker (frequencies of 0% and 7.7% in two Huilliches samples and 4.7% and 5.6% in two Pehuenches samples) (Bravi *et al.*, 2000; Bailliet *et al.*, 2011). The YAP insertion serves as an indicator of non-Native American ancestry, with high probability of African patrilineal ancestry.

In this study, a low African patrilineal contribution, ranging from 2.3% to 14%, and a low frequency of the DYS199T allele (8,5%, indicating low Native American contribution) are suggestive of a mostly European patrilineal origin for the Chilean population studied, as highlighted by Cifuentes *et al.* (2004) in the Santiago population.

Using Native American mtDNA haplogroups to calculate genetic distance by pairwise F_ST_, all observed values were found to be very low (< 5.0%). The largest distances were found between Providencia and Curicó (3.0%) and between Independencia and Curicó (4.7%) communes (Table S1). The fixation index F_ST_ allowed us to evaluate the weight of each haplogroup in the differentiation of populations: no significant difference was found and all observed values were less than 0.9%, which was the value obtained for haplogroup B.

Pairwise F_ST_ calculation using NRY markers showed very low estimates of genetic distance (< 2.4%). The largest genetic distances were found between: Valdivia and Curicó, Puerto Montt and Curicó, and Providencia and Puerto Montt. None of the comparisons showed any significant *p* value, which was indicative of homogeneity among the ancestral patrilineal contributions in these regions (Table S2).

AMOVA results showed a low degree of subdivision in the studied regions. When Native American mtDNA haplogroups were used, 98.6% of the total variation corresponded to differences between individuals within populations, whereas the variation between tested regions (Santiago Metropolitan, Maule, and Los Lagos) was not significantly different from zero. When NRY markers were used, 100% of the total variation observed was within populations (not significant after 1023 permutations), with 1.3% variance among the studied groups (Table S3). This test also showed low φ_CT_ values (variance among groups relative to the total variance).

A potential limitation of this study is the low number of hospitals that were sampled from each region. Hence, we cannot confirm that our sample is representative of the entire region. In addition, we did not study samples from all Chilean regions. Therefore, future studies are still required for a full understanding of the complex genetic origin of the Chilean admixed population.

The strengths of our study include the largest sample size obtained from the Maule (N = 192) and Los Lagos regions (N = 199) and the relatively similar sample sizes among regions. This study also provides a better representation of the populations by using a sample of consecutive births, since blood bank samples are generally more biased with respect to socioeconomic, health-related, and anthropometric variables (Golding *et al.*, 2013). Finally, this study provides information regarding Chilean population structure and contributes to a better understanding of the genetic history of this population, which is important for genetic, medical and anthropological studies.

## Supplementary material

The following online material is available for this article:

Table S1 - Genetic distance (F_ST_) among the seven Chilean communities by means of mtDNA haplogroups.

Table S2 - Genetic distance (FST) among the seven Chilean communities using NRY markers.

Table S3 - AMOVA comparison among the three studied regions.
