# Supplementary material for: Uniparental ancestry markers in Chilean populations
Source: Genet Mol Biol. 2016 Aug 4;39(4):573–9. doi: 10.1590/1678-4685-GMB-2015-0273 (PMC5127147; doi:10.1590/1678-4685-GMB-2015-0273)
Supplement: Supplementary file 1 [file 1415-4757-gmb-1678-4685-GMB-2015-0273-Suppl01.pdf]

Table S1 - Genetic distance (F_{ST}) among the seven Chilean communes by means of mtDNA haplogroups.

	Independência (Santiago MR) [1]	Providencia (Santiago MR) [1]	Santiago (Santiago MR) [1]	Curicó (Maule)	Cauquenes (Maule)	Valdivia (Los Lagos)
Providencia (Santiago MR) [1]	0					
Santiago (Santiago MR) [1]	0	0				
Curicó (Maule)	0.04713*	0.0304*	0.01226			
Cauquenes (Maule)	0.01494	0.00228	0	0		
Valdivia (Los Lagos)	0.02713*	0.01075	0.00296	0.00245	0	
Puerto Montt (Los Lagos)	0.02291*	0.00675	0	0	0	0

[1] Santiago Metropolitan Region

**p* value < 0.05 with 1023 permutations
