# Supplementary material for: Uniparental ancestry markers in Chilean populations
Source: Genet Mol Biol. 2016 Aug 4;39(4):573–9. doi: 10.1590/1678-4685-GMB-2015-0273 (PMC5127147; doi:10.1590/1678-4685-GMB-2015-0273)
Supplement: Supplementary file 2 [file 1415-4757-gmb-1678-4685-GMB-2015-0273-Suppl02.pdf]

Table S2 - Genetic distance (FST) among the seven Chilean communes using NRY markers.

	Independência (Santiago MR) [1]	Providencia (Santiago MR) [1]	Santiago (Santiago MR) [1]	Curicó (Maule)	Cauquenes (Maule)	Valdivia (Los Lagos)
Providencia (Santiago MR) [1]	0					
Santiago (Santiago MR) [1]	0	0				
Curicó (Maule)	0	0	0			
Cauquenes (Maule)	0	0	0	0		
Valdivia (Los Lagos)	0	0.01365	0	0.02269	0	
Puerto Montt (Los Lagos)	0	0.02337	0.00973	0.02146	0	0

All *p* values > 0.05

[1] Santiago Metropolitan Region
