# Supplementary material for: Uniparental ancestry markers in Chilean populations
Source: Genet Mol Biol. 2016 Aug 4;39(4):573–9. doi: 10.1590/1678-4685-GMB-2015-0273 (PMC5127147; doi:10.1590/1678-4685-GMB-2015-0273)
Supplement: Supplementary file 3 [file 1415-4757-gmb-1678-4685-GMB-2015-0273-Suppl03.pdf]

Table S3 - AMOVA comparison among the three studied regions.

		Within Populations		Among populations/Within groups		Among Groups	
Markers	Groups	Variation (%)	*p* value [1]	Variation (%)	*p* value [1]	Variation (%)	*p* value [1]
mtDNA	3	98.64	0.006	0.15	0.303	1.21	0.07
Chromosome Y	3	100.21	0.685	-1.55	0.953	1.35	0.05

[1] *p* value - Significance tests with 1023 permutations
